# Differential glutamine metabolism in the tumor microenvironment – studies in diversity and heterogeneity: A mini-review

**DOI:** 10.3389/fonc.2022.1011191

**Published:** 2022-09-20

**Authors:** Michael D. Claiborne, Robert Leone

**Affiliations:** ^1^ Department of Medicine, Scripps Green Hospital and Scripps Clinic, La Jolla, CA, United States; ^2^ Bloomberg-Kimmel Institute for Cancer Immunotherapy, Sidney Kimmel Comprehensive Cancer Center, Johns Hopkins Medicine, Baltimore, MD, United States

**Keywords:** glutamine, tumor microenvironment, metabolism, cancer, immunooncology

## Abstract

Increased glutamine metabolism is a hallmark of many cancer types. In recent years, our understanding of the distinct and diverse metabolic pathways through which glutamine can be utilized has grown more refined. Additionally, the different metabolic requirements of the diverse array of cell types within the tumor microenvironment complicate the strategy of targeting any particular glutamine pathway as cancer therapy. In this Mini-Review, we discuss recent advances in further clarifying the cellular fate of glutamine through different metabolic pathways. We further discuss potential promising strategies which exploit the different requirements of cells in the tumor microenvironment as it pertains to glutamine metabolism in an attempt to suppress cancer growth and enhance anti-tumor immune responses.

## Introduction

**Figure 1:**
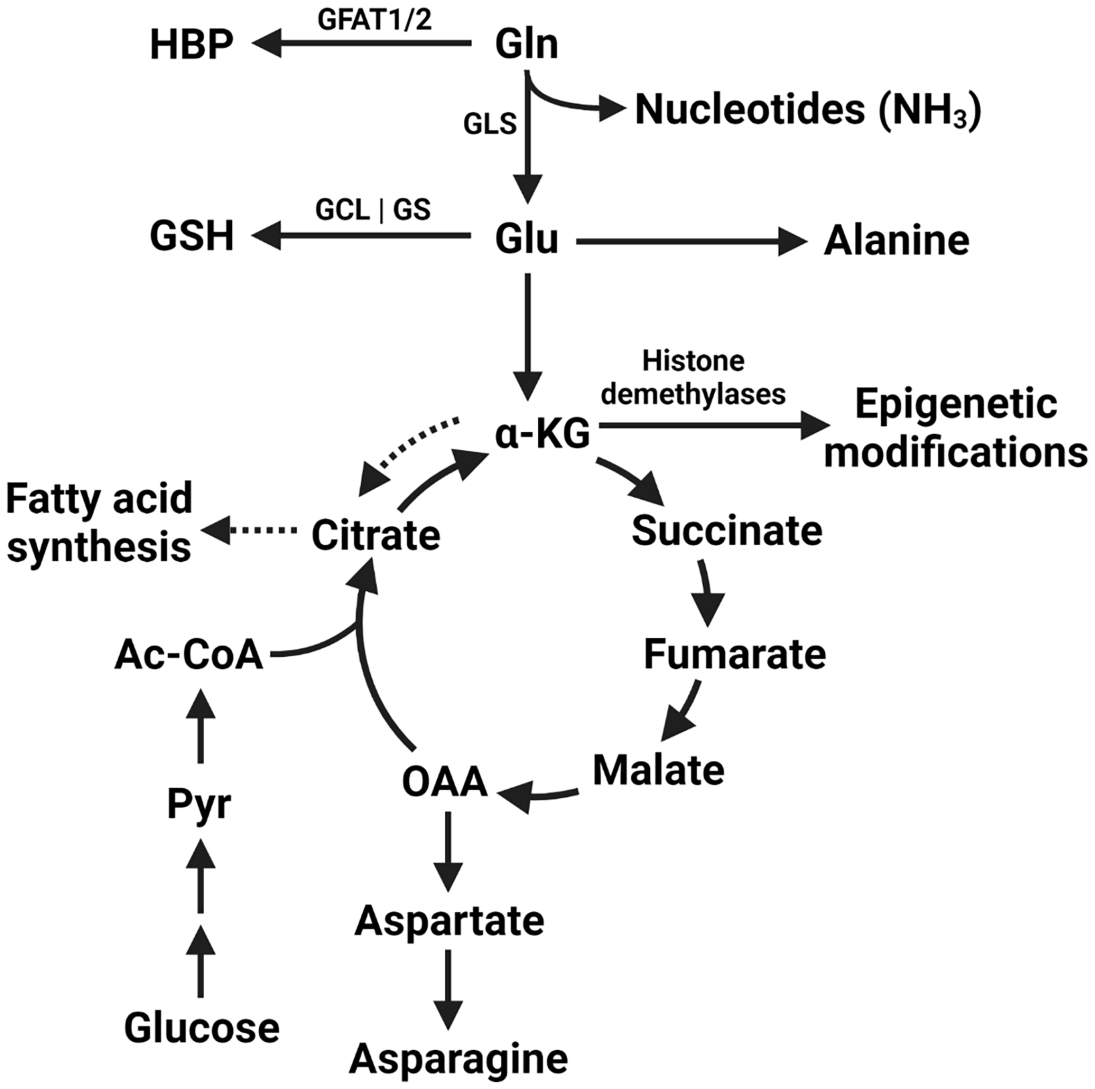
Metabolic fates of glutamine within the tumor cell. The amide nitrogen of glutamine can be utilized by enzymes in pyrimidine and purine nucleotide synthesis. Additionally, glutamine itself is utilized by glutamine-fructose amidotransferase 1/2 (GFAT1/2) to begin the hexosamine biosynthesis pathway (HBP). Glutamine-derived glutamate is utilized in the synthesis of glutathione (GSH) by the subsequent activities of GCL (glutamine-cysteine ligase) and GS (glutathione synthetase). Glutamine is converted to glutamate by the action of glutaminase (GLS) and can be used in the synthesis of alanine or converted to α-KG (alpha-ketoglutarate). Glutamate-derived α-KG can be used as a direct substate for epigenetic modifcation by histone demethylases, as anaplerotic replenishment of the tricarboxylic acid cycle (TCA cycle) intermediates depleted by other biosynthetic reactions, or used for fatty acid synthesis *via* conversion to citrate in the process of reductive carboxylation (dashed arrows). Oxaloacetate (OAA) can additionally be used to synthesise aspartate and asparagine. Glucose-derived pyruvate (Pyr) and acetyl-CoA (Ac-CoA) continue to contribute to the TCA cycle under these anaplerotic conditions.

Cancer cells undergo radical shifts in metabolism to support their growth (1). Increased uptake of glucose by cancer cells with subsequent lactate production irrespective of oxygen availability was described almost 100 years ago (2). Known today as the Warburg effect, this metabolic phenotype is exploited clinically in the form of PET scanning with the glucose analog ^18^F-2-fluoro-2-deoxy-D-glucose (FDG) for cancer staging (3). Recent studies have attempted to extend these findings to other nutrients. Glutamine is the most prevalent amino acid in serum, accounting for over 20% of the circulating amino acid pool (4, 5). Tumor glutamine metabolism is implicated in the synthesis of nucleic acids, the production of glutathione to maintain redox homeostasis, as a source of TCA cycle intermediates, and as a substrate for post-translational modifications, among other pathways (**Figure 1**). Although a nonessential amino acid, glutamine levels can nonetheless have a profound effect on tumor growth. In this regard, a model of “glutamine addiction” has been proposed in which some tumor cells are so dependent on the catabolism of exogenous glutamine that they undergo apoptosis upon its withdrawal (6).

Despite the importance of these processes at a cellular level, the net fate of glutamine as it pertains to an entire tumor is variable and relies on a multitude of factors such as tissue type, specific cellular make-up within a tumor (including immune, stromal, endothelial and cancer cells) and the presence or absence of specific genetic lesions such as mutations in critical proteins such as c-Myc or KRAS. In fact, some tumors display net glutamine catabolism while others display net glutamine synthesis (7, 8). Additional genetic phenotypes which promote glutamine dependence include *VHL* loss in RCC (*via* dependence on reductive carboxylation-driven lipogenesis) and loss of the negative NRF2 regulator *KEAP1* driving glutamine dependence in lung cancers (9, 10). Furthermore, the supplementation requirement of exogenous glutamine for certain cancer cells grown in culture does not necessarily extrapolate to their metabolic requirements *in vivo* (11). Even with the tremendous importance of glutamine to the metabolism of cancer cells, promising preclinical results using targeted approaches to specific glutamine metabolic pathways have not always translated to success in the clinic (12, 13). In this review, we discuss these seemingly paradoxical findings and highlight new studies that have added to our understanding of the adaptability of tumor metabolism. We discuss how interdependence between cancer cells and other cells in the tumor microenvironment (TME) may render metabolic inhibition of particular pathways ineffective. As such, we emphasize how an understanding of the activity of multiple metabolic programs across diverse cell types in a tumor may yield more comprehensive information about utilization of critical metabolites such as glutamine. It is within this context that we suggest that the approach to glutamine inhibition should ideally be tailored based on the specific genetic lesions and cellular make-up of the TME. As such, this approach will invoke a therapeutic middle ground between broad metabolic inhibition, with incumbent risks of adverse effects from normal tissue cytotoxicity, and highly targeted inhibition of specific pathways, an approach that could restrict effects to only a subset of cells in the tumor microenvironment allowing for adaptation and progression of malignant tissues.

## The nature of cellular metabolic interdependence

Quantification of metabolic flux relies on accurate metabolite identification through mass spectrometry (MS) or nuclear magnetic resonance (NMR) analysis and can be broadly classified as targeted (searching for known metabolites) or non-targeted (a discovery-based approach). Targeted analyses rely on large high-quality internal compound standard databases for reference, while non-targeted analyses create large high-dimensional data sets that require multivariate analysis for appropriate interpretation (14). Despite advances in metabolite identification, software packages for statistical analysis, and cell culture techniques that have facilitated this advanced study of metabolic pathways in monoculture, metabolism remains a complex and multifaceted process. Metabolism in a living organism is a constantly changing interplay of a multitude of cell types that alter flux through pathways depending on signals in their microenvironment (15). The importance of microenvironment in terms of glutamine metabolism is underlined by a study by Davidson, et al., which demonstrated that cultured cells that rely on glutamine catabolism *in vitro* do not necessarily require glutamine for growth *in vivo*, as murine oncogenic KRAS-driven lung cancer cells do not extensively utilize glutamine-derived carbons in TCA cycle reactions (11). The authors of this study hypothesize that this may be due in part to the differences in cellular microenvironment that fundamentally alter both metabolite and waste utilization.

**Figure 2:**
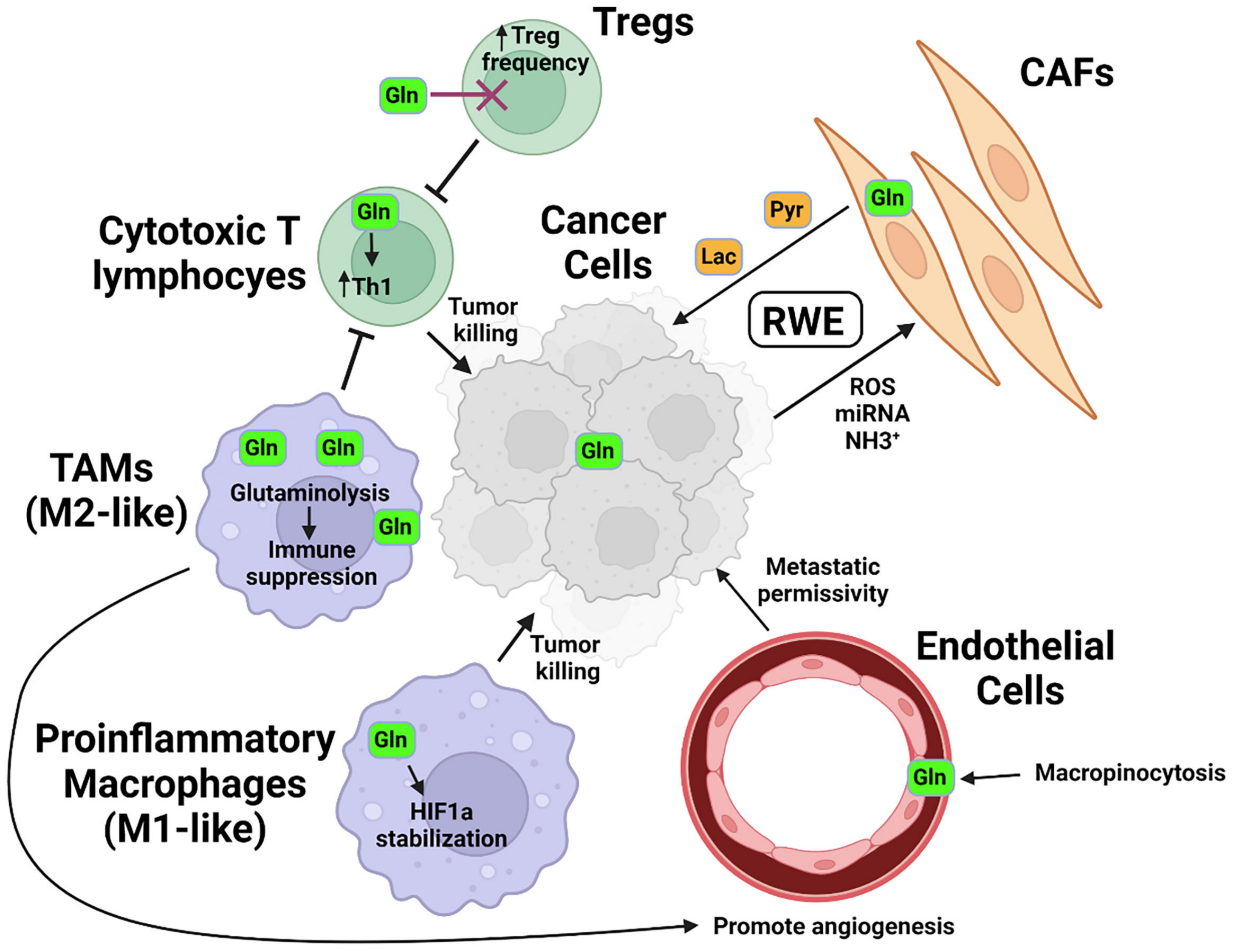
Glutamine in the tumor microenvironment. In response to signals including ROS and miRNA secreted by tumor cells and utilizing metabolites such as tumor-derived NH3^+^, cancer associated fibroblasts (CAFs) metabolize glutamine into high-energy intermediate molecules such as pyruvate (Pyr) or lactate (Lac), which are then secreted and utilized by tumor cells. Glutamine is metabolized by pro-inflammatory, or M1-like macrophages into succinate, which helps stabilize HIF1a and drives expression of pro-inflammatory and tumoricidal genes. In tumor-associated macrophages (TAMs), or M2-like macrophages, glutamine is metabolized to α-KG and serves as a co-factor for epigenetic modification by histone demethylases which support an anti-inflammatory phenotype. These macrophages additionally modulate the activity of vascular endothelial cells, aiding in the remodeling that permits for tumor metastasis *via* vascular egress. T lymphocytes also display differential glutamine requirements, with immunosuppressive T-regulatory, or Treg frequencies increased in the absence of glutamine and glutamine supporting the development of pro-inflammatory Th1-phenotype cells.

The effect of the microenvironment on metabolic programming of specific cellular constituents therefore can in part be reflective of the metabolic interdependence, or co-metabolism, of specific resident cell types. The notion that non-cancerous cells in the tumor microenvironment contribute to cancer cell metabolism and growth has been particularly well studied in the case of glucose utilization and has led to the development of the concept termed the “reverse Warburg effect”, or RWE (**Figure 2**). In this model, cancer cells induce aerobic glycolysis in cancer-associated fibroblasts (CAFs) through oxidative stress, exosomal microRNAs, or signaling modalities such as TGF-β (16–18). The CAFs in turn produce and secrete high-energy intermediates from aerobic glycolysis, such as pyruvate and lactate, which conversely support oxidative phosphorylation in the tumor cells (19, 20). Interdependence centered on glutamine metabolism in cell types sharing a microenvironment is particularly well demonstrated in physiological processes such as brain cell metabolism, wherein glutamate secreted by neurons as an excitatory neurotransmitter undergoes rapid uptake and conversion by astrocytes into glutamine using enzymes not expressed in neurons themselves (21). Through this mechanism, cell type-specific metabolism tightly regulates the process of neurotransmission.

In addition to known physiological processes, an increasing body of evidence implicates cellular interdependence in glutamine metabolism during pathophysiological processes such as tumorigenesis. Indeed, studies in animal breast cancer models demonstrated that glutamine-derived ammonia, thought to be a simple waste product of cancer cell metabolism, was recycled by CAFs into other amino acids which could serve as a critical nitrogen source fueling tumorigenesis (22). Addressing similar findings, Yang et al. found that by inhibiting both cellular constituents in this exchange, a combination strategy targeting glutamine synthetase in fibroblasts alongside glutaminase in cancer cells reduced tumor weight and metastatic burden in orthotopic mouse ovarian cancer models (23). Interestingly, recent computational modeling has also challenged the notion that tumor cells are reliant on exogenous glutamine for growth, as glutamine-derived carbon did not impact *in silico* growth models, which remained limited by glucose and oxygen under various modeling conditions (24).

Future work aimed at comprehensive assessment of the metabolism of the entire tumor, which includes cancer cells, stromal cells, and infiltrating immune cells, will likely be critical in order to achieve meaningful therapeutic outcomes. We next focus on different metabolic fates of glutamine within the tumor microenvironment and attempts to exploit the differential metabolic requirements of cells therein.

## Anaplerosis

Conversion of glutamine to glutamate *via* glutaminase (GLS) with subsequent conversion to alpha ketoglutarate (α-KG) allows for entry of glutamine-derived carbons into the TCA cycle to replenish intermediates that have been extracted for other biosynthetic reactions (25). Glutaminase therefore serves as a logical target for inhibition of tumor metabolism, and preclinical studies of the glutaminase inhibitor CB-839 demonstrated efficacy in slowing tumor cell growth (26–28). Unfortunately, successful clinical application has been more challenging (12, 13). The clinical shortcomings of glutaminase inhibition to slow tumor growth may be based in the adaptable nature of tumor metabolism. For example, asparagine can also serve an anaplerotic role as a source of oxaloacetate, and tumor asparagine synthetase is sufficient to prevent apoptosis in cancer cells deprived of glutamine (29). The availability of other nutrients can also control the degree to which glutamine is utilized for anaplerotic reactions. Muir et al. recapitulated the aforementioned decrease in glutamine utilization seen *in vivo* with cancer cells cultured in bovine serum instead of cell culture media, which more closely approximates physiological nutrient levels (30). Levels of the amino acid cystine alone were sufficient to explain these findings, as cystine activated the cystine/glutamate antiporter xCT (SLC7A11) to drive tumor glutamine uptake. The authors hypothesize that the supraphysiological levels of glutamine used in most cell culture media led to increased anaplerotic utilization and may have been responsible for some of the antiproliferative effects seen following glutaminase inhibition. However, they propose that co-administration of cystine with glutaminase inhibitors could comprise a reasonable therapeutic modality, with cystine driving glutamine uptake and utilization rendering tumors sensitive to glutaminase inhibition. Rossiter et al. additionally demonstrated pyruvate to be a conditional suppressor of glutamine dependence in K562 cells through CRISPR screens using relatively glutamine-rich standard culture medium versus HPLM (human plasma-like medium) with more physiological glutamine levels, wherein pyruvate supplementation rescued growth defects seen in GLS-deficient cells in standard culture media and withdrawal of pyruvate in HPLM created similar growth defects which were dependent on α-KG production (31).

A broader blockade of cellular glutamine programs has been explored with glutamine antimetabolites, most notably 6-diazo-5-oxo-L-norleucine (DON), which inhibits not only glutaminase but a range of enzymatic reactions utilizing glutamine as a substrate (32). Likely related to its broad mechanism of inhibition, early clinical trials using DON showed a narrow therapeutic index despite signs of antitumor efficacy (33). The development of a DON prodrug with protecting groups preferentially cleaved to release DON in tumor tissue over serum has permitted for further animal study of this antimetabolite approach. Pharmacokinetics of this prodrug revealed that although T_max_ for detectable DON was delayed in tumor versus serum (30 minutes versus 5 minutes), C_max_ was over 20% higher in the tumor with more than double the AUC value for detectable DON tumor versus serum over the course of administration (34).

Preclinical studies with this DON prodrug demonstrate a decrease in tumor oxidative phosphorylation and ATP generation. Notably, although dividing T lymphocytes are also among the cells known to utilize glutamine for Warburg metabolism at activation, DON did not inhibit tumor infiltrating lymphocytes (TILs) but rather endowed them with a highly-activated, long-lived phenotype. Cell type-specific differences in metabolic compensation appear to be critical to this finding, such as the ability of TILs but not tumor cells to preferentially utilize pyruvate *via* pyruvate carboxylase (PC) for replenishment of TCA cycle intermediates (e.g., oxaloacetate) under conditions of glutamine blockade. Further studies suggested an additional mechanism of tumor control arose from alteration in myeloid-derived suppressor cell (MDSC) phenotype under glutamine antimetabolite therapy, with these typically pro-tumorigenic cells converting to a more pro-inflammatory phenotype while losing expression of IDO, the enzyme responsible for synthesizing the immunosuppressive metabolite kynurenine (35). These pre-clinical studies provided evidence to initiate a Phase 1 and Phase 2a first-in-human study of this DON pro-drug, now known as DRP-104, for patients with advanced solid tumors (NCT04471415). These studies highlight the potential of exploiting differential cell-specific metabolic requirements within the tumor microenvironment, circumventing the ability of cancerous cells to utilize their metabolic flexibility to overcome simple nutrient deprivation.

## The hexosamine biosynthesis pathway

Another role of glutamine is fueling, along with glucose, the hexosamine biosynthesis pathway, or HBP, which provides the necessary glycosaminoglycans for extracellular matrix (ECM) synthesis and intracellular glycosylation reactions (36). The rate-limiting step in this pathway, glutamine-fructose amidotransferase 1/2 (GFAT1/2), condenses glutamine with glucose-derived fructose-6-phosphate (F6P) to ultimately synthesize uridine diphosphate N-acetylglucosamine (UDP-GlcNAc). Enzymes such as O-GlcNAc transferase (OGT) utilize UDP-GlcNAc to glycosylate target proteins, while the hyaluronan synthases (HAS1-3) utilize this UDP-GlcNAc for ECM synthesis. Recent studies targeting the HBP demonstrated that glutamine blockade with DON decreases ECM component synthesis and alters ECM structure in pancreatic cancer models, shifting the tumor microenvironment from immune-exclusionary to one more amenable to immune cell infiltration (37). As such, DON treatment sensitized tumors to checkpoint blockade therapy. Control of tumor burden in this model was dependent on the ability of T lymphocytes to access and kill tumor cells.

Further studies have demonstrated additional cancer cell-intrinsic mechanisms by which tumor cells rely on the HBP for progression and metastasis and the therapeutic potential of HBP inhibition. Rossi et al. describe a correlation between loss of phosphoglycerate dehydrogenase (PHGDH) expression in primary tumors of breast cancer patients and shortened metastasis-free survival. In mice, the authors demonstrated that PHGDH interacts with phosphofructokinase, and the loss of this interaction increases flux through hexosamine biosynthesis (38). This increased flux provides the substrate for integrin α_V_β_3_ sialylation, which facilitates tumor metastatic activity. Studies utilizing direct HBP inhibition have also demonstrated reduction in pancreatic patient-derived tumor xenograft outgrowths with genetic ablation or pharmacologic inhibition of the HBP enzyme phosphoacetylglucosamine mutase 3 (PGM3) (39). These studies support the notion that targeting metabolic pathways utilized by the malignant population of the tumor microenvironment (tumor cells) while seemingly not utilized to such a degree by other populations (such as T lymphocytes, which are not known to construct extensive ECM) could serve as an exploitable target for metabolic therapy of established solid tumors.

## Epigenetic modification

Glutamine-derived α-KG is a critical cofactor utilized by a family of histone demethylases to control gene expression of many cancer-promoting genes (40). These regulated genes differ greatly across tumor types and some members of this family of demethylases, such as Jmjd3, can be thought of as oncogenes or tumor suppressor genes depending on the cellular context (41–43). In addition to tumor type, the regional availability of glutamine within a tumor can also directly affect epigenetic modification and control of cellular differentiation. Pan et al. demonstrated differential histone methylation in glutamine-rich peripheral tumor compared to the glutamine-depleted tumor core in BRAF V600E tumor models (44). This difference in glutamine levels was reflected in the tumor epigenetic landscape, with an increase in H3K27 methylation driven by low glutamine in the core of the tumor leading to an increase in expression of dedifferentiation markers and an increased resistance to BRAF inhibitor treatment. As hypoxia (as is often encountered in the TME) inhibits both histone and DNA demethylation (45), it is reasonable to query whether glutamine availability additionally affects DNA modifying enzymes in a similar manner. Indeed, ten-eleven translocation (TET) enzyme-mediated locus-specific CpG demethylation decreased in chicken embryos treated with the glutaminase inhibitor bis-2-(5-phenylacetamido-1,3,4-thiadiazol-2-yl) ethyl sulfide (BPTES) under dynamic conditions of TET induction, although it should be noted these studies were not conducted in the setting of cancer (46).

Additional studies have shed light on how glutamine metabolism can control the epigenetic landscape of other critical cell populations within a tumor. Glutamine metabolism differentially affected T lymphocyte subsets in work by Johnson et al., with chromatin accessibility and gene expression altered to differentially restrict Th17 but promote Th1 development under conditions of glutaminase deficiency or inhibition (47). Glutaminase activity is also critical in the transduction of IL-2 signaling into differentiation programs that control CD8+ T lymphocyte memory or effector fate (48). In myeloid cells, Liu et al. demonstrated that glutaminolysis-derived α-KG is critical for Jmjd3-dependent epigenetic modification and activation of gene programs more associated with the immunosuppressive M2 phenotype as compared to the more pro-inflammatory M1 phenotype (49). Macrophages and other myeloid cells make up a sizeable portion of the tumor microenvironment (**Figure 2**), and their phenotype plays a major role in tumor progression and response to therapy (50). As glutamine metabolism impacts multiple cell types in the immune compartment of the tumor microenvironment, informed selection of which tumors to treat with epigenetic-targeting drugs that rely on glutamine metabolism will be critical in determining response to therapy.

## Redox homeostasis

Glutamine-derived glutamate is used in the synthesis of glutathione (GSH), a tripeptide of glutamate, cysteine, and glycine which serves as a major cytosolic antioxidant in eukaryotes (51). Tumor cells generate extensive free radicals, and a significant portion of extracellular glutamine has been shown to be utilized in glutathione synthesis for maintenance of redox homeostasis in lung cancer models (52). However, as is the case with most metabolic pathways, tumor cells are adaptable in the context of glutamine withdrawal. Sun et al. demonstrated that glutamine withdrawal activates the serine-glycine one-carbon (SGOC) metabolism, facilitating the diversion of glycolytic intermediates to serine and glycine synthesis (53). This supports not only GSH synthesis but *de novo* nucleotide synthesis *via* the production of methylation substrates from SGOC that are critical for thymidine synthesis. Successful targeting of glutamine metabolism for perturbation of redox homeostasis will therefore rely on a more complete understanding of the metabolic requirements of all cell types within a tumor. Indeed, preclinical studies have shown that glutaminase inhibition can impair GSH synthesis and lead to oxidative stress-induced death in multiple types of AML, synergizing with other drugs that disrupt redox state (54). Further studies have demonstrated that the glutamine transporter inhibitor V-9302 selectively blocked glutamine uptake by cancer cells, but not CD8+ T lymphocytes, in preclinical breast cancer models (55). Glutathione production and cytotoxic capability was increased in T lymphocytes under these conditions as a result of a T lymphocyte-specific compensatory upregulation of the amino acid transporter SLC6A14, which allowed for increased synthesis of glutathione that tumor cells were unable to parallel. Similar to the aforementioned studies of DON prodrugs, a cell-type specific approach whereby differential metabolism is exploited between tumor and non-tumor cells may facilitate the approach of metabolic therapies as they pertain to redox homeostasis.

## Nucleotide synthesis

Glutamine plays a critical role in the synthesis of purine and pyrimidine nucleotides both as a direct nitrogen donor and *via* anaplerotic reactions that replenish amino acids used in nucleotide synthesis (56). As mentioned earlier, Sun et al. demonstrated that increased *de novo* serine and glycine synthesis in tumor cells under conditions of glutamine withdrawal facilitates the necessary synthesis of nucleic acids *via* increased flux through one-carbon metabolism, suggestive of metabolic flexibility of cancer cells in terms of nucleic acid metabolism (53). Interestingly, this inherent adaptability is evident even within subtypes of the same cancer, as analyses of small cell lung cancers has revealed a subset dependent on Myc-driven inosine monophosphate dehydrogenase (IMPDH) activity specifically for ribosomal RNA (rRNA) synthesis over mRNA synthesis, suggesting multiple avenues of metabolic adaptation as it pertains to nucleotide metabolism beyond the need for synthesis of genomic DNA (57).

The contribution of glutamine to cellular nucleic acid synthesis may represent the most generalizable and least cell-type specific modulation of metabolism across cancers. A large-scale proteomics approach analyzing a multitude of tumor types has demonstrated an almost-universal shift of glutamine as a source for anaplerotic reactions of the TCA cycle and towards enzymes in nucleic acid synthesis such as phosphoribosyl pyrophosphate amidotransferase (PPAT) during cancer progression (58, 59). In fact, in these analyses, PPAT activity bore the strongest prognostic value of any metabolic enzyme. Nevertheless, wholesale blockade of this pathway could have consequences on other cell types, as glutamine-dependent nucleotide synthesis in immune cells has been demonstrated as critical not only for replication of genomic DNA during cell division but also to support rRNA synthesis and ribosomal biogenesis (60, 61). That said, whether or not co-metabolic processes, such as those between cancer cells and immune cells, for instance, could compensate and allow for an asymmetric targeting of nucleic acid metabolism in distinct cellular compartments within a tumor is yet to be determined. For instance, could cancer cells that are dying in response to glutamine blockade provide a source of nucleic acid building blocks to fuel salvage pathways of responding immune cells? Such an approach would necessarily exploit a differential plasticity between cancer cells and immune cells.

## Conclusion

Alterations in tumor metabolism have attracted scientific attention for over a century. While modern approaches have demonstrated how metabolites are utilized in exquisite detail, recent studies suggest that the heterogeneous nature of the tumor microenvironment, the interconnected nature of cellular metabolism, and the plasticity of intracellular metabolic pathways create significant hurdles to successful application of targeting a single, highly specific glutamine metabolic pathway such as glutaminase activity. Evidence of competition for glucose and differential glucose metabolism between cells within the tumor microenvironment has existed for some time (62). We now have evidence that glutamine can also be utilized in a heterogeneous manner within the TME. Frameworks such as the RWE can help us to rethink approaches to metabolic inhibition in cancer therapy, while other cell types within the TME, such as endothelial cells (63–65) and stromal cells (66), in addition to the immune cells already discussed, can be highly dependent upon glutamine metabolism (**Figure 2**). Understanding more completely which cells are utilizing glutamine for which pathways may permit for targeted therapies that can realize the full potential of inhibiting this critical metabolite in cancer.

## Author contributions

MDC wrote the manuscript and created the figures. RL revised the manuscript and provided scientific insight regarding literature selection.

## Funding

The study was funded by the following: The Bloomberg-Kimmel Institute for Cancer Immunotherapy, R01CA226765 (Robert Leone - Role: PI/PD) R01CA229451 (Robert Leone - Role: Key Personnel).

## Conflict of interest

MDC declares no conflicts of interest. RL is an inventor on JHU patents covering novel glutamine antagonist prodrugs. These patents have been licensed to Dracen Pharmaceuticals, Inc.

## Publisher’s note

All claims expressed in this article are solely those of the authors and do not necessarily represent those of their affiliated organizations, or those of the publisher, the editors and the reviewers. Any product that may be evaluated in this article, or claim that may be made by its manufacturer, is not guaranteed or endorsed by the publisher.

## References

[1] DeBerardinis RJ Chandel NS. Fundamentals of cancer metabolism. Sci Adv (2016) 2(5):e1600200. doi: 10.1126/sciadv.1600200 27386546 PMC4928883

[2] Warburg O. Über den Stoffwechsel der Carcinomzelle. Naturwissenschaften, 12, 1131–1137 (1924). doi: 10.1007/BF01504608

[3] Griffeth LK. Use of PET/CT scanning in cancer patients: technical and practical considerations. Proc (Bayl Univ Med Cent) (2005) 18(4):321–30. doi: 10.1080/08998280.2005.11928089 PMC1255942 16252023

[4] Roth E. Nonnutritive effects of glutamine. J Nutr (2008) 138(10):2025S–31S. doi: 10.1093/jn/138.10.2025S 18806119

[5] Mayers JR Vander Heiden MG. Famine versus feast: understanding the metabolism of tumors *in vivo* . Trends Biochem Sci (2015) 40(3):130–40. doi: 10.1016/j.tibs.2015.01.004 PMC4340757 25639751

[6] Yang L Venneti S Nagrath D. Glutaminolysis: A hallmark of cancer metabolism. Annu Rev BioMed Eng (2017) 19:163–94. doi: 10.1146/annurev-bioeng-071516-044546 28301735

[7] Yuneva MO Fan TW Allen TD Higashi RM Ferraris DV Tsukamoto T. The metabolic profile of tumors depends on both the responsible genetic lesion and tissue type. Cell Metab (2012) 15(2):157–70. doi: 10.1016/j.cmet.2011.12.015 PMC3282107 22326218

[8] Dejure FR Eilers M. MYC and tumor metabolism: chicken and egg. EMBO J (2017) 36(23):3409–20. doi: 10.15252/embj.201796438 PMC5709748 29127156

[9] Gameiro PA Yang J Metelo AM Pérez-Carro R Baker R Wang Z. *In vivo* HIF-mediated reductive carboxylation is regulated by citrate levels and sensitizes VHL-deficient cells to glutamine deprivation. Cell Metab (2013) 17(3):372–85. doi: 10.1016/j.cmet.2013.02.002 PMC4003458 23473032

[10] Romero R Sayin VI Davidson SM Bauer MR Singh SX LeBoeuf SE. Keap1 loss promotes kras-driven lung cancer and results in dependence on glutaminolysis. Nat Med (2017) 23(11):1362–8. doi: 10.1038/nm.4407 PMC5677540 28967920

[11] Davidson SM Papagiannakopoulos T Olenchock BA Heyman JE Keibler MA Luengo A. Environment impacts the metabolic dependencies of ras-driven non-small cell lung cancer. Cell Metab (2016) 23(3):517–28. doi: 10.1016/j.cmet.2016.01.007 PMC4785096 26853747

[12] Phase 2 CANTATA study of telaglenastat fails to meet primary end point for advanced clear cell RCC (2021). Available at: https://www.cancernetwork.com/view/phase-2-cantata-study-of-telaglenastat-fails-to-meet-primary-end-point-for-advanced-clear-cell-rcc.

[13] Calithera biosciences announces decision to discontinue KEAPSAKE clinical trial (2021). Available at: https://www.biospace.com/article/releases/calithera-biosciences-announces-decision-to-discontinue-keapsake-clinical-trial/.

[14] Saoi M Britz-McKibbin P. New advances in tissue metabolomics: A review. Metabolites (2021) 11(10):672. doi: 10.3390/metabo11100672 34677387 PMC8541552

[15] Goodpaster BH Sparks LM. Metabolic flexibility in health and disease. Cell Metab (2017) 25(5):1027–36. doi: 10.1016/j.cmet.2017.04.015 PMC5513193 28467922

[16] Pavlides S Whitaker-Menezes D Castello-Cros R Flomenberg N Witkiewicz AK Frank PG. The reverse warburg effect: aerobic glycolysis in cancer associated fibroblasts and the tumor stroma. Cell Cycle (2009) 8(23):3984–4001. doi: 10.4161/cc.8.23.10238 19923890

[17] Martinez-Outschoorn UE Balliet RM Rivadeneira DB Chiavarina B Pavlides S Wang C. Oxidative stress in cancer associated fibroblasts drives tumor-stroma co-evolution: A new paradigm for understanding tumor metabolism, the field effect and genomic instability in cancer cells. Cell Cycle (2010) 9(16):3256–76. doi: 10.4161/cc.9.16.12553 PMC3041164 20814239

[18] Scognamiglio I Cocca L Puoti I Palma F Ingenito F Quintavalle C. Exosomal microRNAs synergistically trigger stromal fibroblasts in breast cancer. Mol Ther Nucleic Acids (2022) 28:17–31. doi: 10.1016/j.omtn.2022.02.013 35317202 PMC8908025

[19] Yan W Wu X Zhou W Fong MY Cao M Liu J. Cancer-cell-secreted exosomal miR-105 promotes tumour growth through the MYC-dependent metabolic reprogramming of stromal cells. Nat Cell Biol (2018) 20(5):597–609. doi: 10.1038/s41556-018-0083-6 29662176 PMC5920728

[20] Sakamoto A Kunou S Shimada K Tsunoda M Aoki T Iriyama C. Pyruvate secreted from patient-derived cancer-associated fibroblasts supports survival of primary lymphoma cells. Cancer Sci (2019) 110(1):269–78. doi: 10.1111/cas.13873 PMC6317936 30426593

[21] Tsacopoulos M Magistretti PJ. Metabolic coupling between glia and neurons. J Neurosci (1996) 16(3):877–85. doi: 10.1523/JNEUROSCI.16-03-00877.1996 PMC6578818 8558256

[22] Spinelli JB Yoon H Ringel AE Jeanfavre S Clish CB Haigis MC. Metabolic recycling of ammonia *via* glutamate dehydrogenase supports breast cancer biomass. Science (2017) 358(6365):941–6. doi: 10.1126/science.aam9305 PMC5748897 29025995

[23] Yang L Achreja A Yeung TL Mangala LS Jiang D Han C. Targeting stromal glutamine synthetase in tumors disrupts tumor microenvironment-regulated cancer cell growth. Cell Metab (2016) 24(5):685–700. doi: 10.1016/j.cmet.2016.10.011 27829138 PMC7329194

[24] Shan M Dai D Vudem A Varner JD Stroock AD. Multi-scale computational study of the warburg effect, reverse warburg effect and glutamine addiction in solid tumors. PloS Comput Biol (2018) 14(12):e1006584. doi: 10.1371/journal.pcbi.1006584 30532226 PMC6285468

[25] Owen OE Kalhan SC Hanson RW. The key role of anaplerosis and cataplerosis for citric acid cycle function. J Biol Chem (2002) 277(34):30409–12. doi: 10.1074/jbc.R200006200 12087111

[26] Parlati F Bromley-Dulfano S Demo S Janes J Gross M Lewis E. Antitumor activity of the glutaminase inhibitor CB-839 in hematological malignances. Blood (2013) 122(21):4226. doi: 10.1182/blood.V122.21.4226.4226

[27] Lee P Malik D Perkons N Huangyang P Khare S Rhoades S. Targeting glutamine metabolism slows soft tissue sarcoma growth. Nat Commun (2020) 11(1):498. doi: 10.1038/s41467-020-14374-1 31980651 PMC6981153

[28] Thompson RM Dytfeld D Reyes L Robinson RM Smith B Manevich Y. Glutaminase inhibitor CB-839 synergizes with carfilzomib in resistant multiple myeloma cells. Oncotarget (2017) 8(22):35863–76. doi: 10.18632/oncotarget.16262 PMC5482623 28415782

[29] Zhang J Fan J Venneti S Cross JR Takagi T Bhinder B. Asparagine plays a critical role in regulating cellular adaptation to glutamine depletion. Mol Cell (2014) 56(2):205–18. doi: 10.1016/j.molcel.2014.08.018 PMC4224619 25242145

[30] Muir A Danai LV Gui DY Waingarten CY Lewis CA Vander Heiden MG. Environmental cystine drives glutamine anaplerosis and sensitizes cancer cells to glutaminase inhibition. Elife (2017) 6:e27713. doi: 10.7554/eLife.27713 28826492 PMC5589418

[31] Rossiter NJ Huggler KS Adelmann CH Keys HR Soens RW Sabatini DM. CRISPR screens in physiologic medium reveal conditionally essential genes in human cells. Cell Metab (2021) 33(6):1248–63.e9. doi: 10.1016/j.cmet.2021.02.005 33651980 PMC8172426

[32] Thangavelu K Chong QY Low BC Sivaraman J. Structural basis for the active site inhibition mechanism of human kidney-type glutaminase (KGA). Sci Rep (2014) 4:3827. doi: 10.1038/srep03827 24451979 PMC4929687

[33] Ahluwalia GS Grem JL Hao Z Cooney DA. Metabolism and action of amino acid analog anti-cancer agents. Pharmacol Ther (1990) 46(2):243–71. doi: 10.1016/0163-7258(90)90094-I 2108451

[34] Leone RD Zhao L Englert JM Sun IM Oh MH Sun IH. Glutamine blockade induces divergent metabolic programs to overcome tumor immune evasion. Science (2019) 366(6468):1013–21. doi: 10.1126/science.aav2588 PMC7023461 31699883

[35] Oh MH Sun IH Zhao L Leone RD Sun IM Xu W. Targeting glutamine metabolism enhances tumor-specific immunity by modulating suppressive myeloid cells. J Clin Invest (2020) 130(7):3865–84. doi: 10.1172/JCI131859 PMC7324212 32324593

[36] Akella NM Ciraku L Reginato MJ. Fueling the fire: emerging role of the hexosamine biosynthetic pathway in cancer. BMC Biol (2019) 17(1):52. doi: 10.1186/s12915-019-0671-3 31272438 PMC6610925

[37] Sharma NS Gupta VK Garrido VT Hadad R Durden BC Kesh K. Targeting tumor-intrinsic hexosamine biosynthesis sensitizes pancreatic cancer to anti-PD1 therapy. J Clin Invest (2020) 130(1):451–65. doi: 10.1172/JCI127515 PMC6934212 31613799

[38] Rossi M Altea-Manzano P Demicco M Doglioni G Bornes L Fukano M. PHGDH heterogeneity potentiates cancer cell dissemination and metastasis. Nature (2022) 605(7911):747–53. doi: 10.1038/s41586-022-04758-2 PMC9888363 35585241

[39] Ricciardiello F Gang Y Palorini R Li Q Giampà M Zhao F. Hexosamine pathway inhibition overcomes pancreatic cancer resistance to gemcitabine through unfolded protein response and EGFR-akt pathway modulation. Oncogene (2020) 39(20):4103–17. doi: 10.1038/s41388-020-1260-1 32235891

[40] Xiang Y Zhu Z Han G Lin H Xu L Chen CD. JMJD3 is a histone H3K27 demethylase. Cell Res (2007) 17(10):850–7. doi: 10.1038/cr.2007.83 17923864

[41] Hong Z Li H Li L Wang W Xu T. Different expression patterns of histone H3K27 demethylases in renal cell carcinoma and bladder cancer. Cancer biomark (2017) 18(2):125–31. doi: 10.3233/CBM-160003 PMC13020585 27983522

[42] Tang B Qi G Tang F Yuan S Wang Z Liang X. Aberrant JMJD3 expression upregulates slug to promote migration, invasion, and stem cell-like behaviors in hepatocellular carcinoma. Cancer Res (2016) 76(22):6520–32. doi: 10.1158/0008-5472.CAN-15-3029 27651311

[43] Xu Z Xia Y Xiao Z Jia Y Li L Jin Y. Comprehensive profiling of JMJD3 in gastric cancer and its influence on patient survival. Sci Rep (2019) 9(1):868. doi: 10.1038/s41598-018-37340-w 30696880 PMC6351656

[44] Pan M Reid MA Lowman XH Kulkarni RP Tran TQ Liu X. Regional glutamine deficiency in tumours promotes dedifferentiation through inhibition of histone demethylation. Nat Cell Biol (2016) 18(10):1090–101. doi: 10.1038/ncb3410 PMC5536113 27617932

[45] Yoo HC Yu YC Sung Y Han JM. Glutamine reliance in cell metabolism. Exp Mol Med (2020) 52(9):1496–516. doi: 10.1038/s12276-020-00504-8 PMC8080614 32943735

[46] Rosenberg T Kisliouk T Cramer T Shinder D Druyan S Meiri N. Embryonic heat conditioning induces TET-dependent cross-tolerance to hypothalamic inflammation later in life. Front Genet (2020) 11:767. doi: 10.3389/fgene.2020.00767 32849788 PMC7419591

[47] Johnson MO Wolf MM Madden MZ Andrejeva G Sugiura A Contreras DC. Distinct regulation of Th17 and Th1 cell differentiation by glutaminase-dependent metabolism. Cell (2018) 175(7):1780–95. doi: 10.1016/j.cell.2018.10.001 PMC6361668 30392958

[48] Chisolm DA Savic D Moore AJ Ballesteros-Tato A León B Crossman DK. CCCTC-binding factor translates interleukin 2- and α-Ketoglutarate-Sensitive metabolic changes in T cells into context-dependent gene programs. Immunity (2017) 47(2):251–67.e7. doi: 10.1016/j.immuni.2017.07.015 28813658 PMC5654635

[49] Liu PS Wang H Li X Chao T Teav T Christen S. α-ketoglutarate orchestrates macrophage activation through metabolic and epigenetic reprogramming. Nat Immunol (2017) 18(9):985–94. doi: 10.1038/ni.3796 28714978

[50] Zhou J Tang Z Gao S Li C Feng Y Zhou X. Tumor-associated macrophages: Recent insights and therapies. Front Oncol (2020) 10:188. doi: 10.3389/fonc.2020.00188 32161718 PMC7052362

[51] Pompella A Visvikis A Paolicchi A De Tata V Casini AF. The changing faces of glutathione, a cellular protagonist. Biochem Pharmacol (2003) 66(8):1499–503. doi: 10.1016/S0006-2952(03)00504-5 14555227

[52] Sappington DR Siegel ER Hiatt G Desai A Penney RB Jamshidi-Parsian A. Glutamine drives glutathione synthesis and contributes to radiation sensitivity of A549 and H460 lung cancer cell lines. Biochim Biophys Acta (2016) 1860(4):836–43. doi: 10.1016/j.bbagen.2016.01.021 PMC4768472 26825773

[53] Sun L Song L Wan Q Wu G Li X Wang Y. cMyc-mediated activation of serine biosynthesis pathway is critical for cancer progression under nutrient deprivation conditions. Cell Res (2015) 25(4):429–44. doi: 10.1038/cr.2015.33 PMC4387561 25793315

[54] Gregory MA Nemkov T Park HJ Zaberezhnyy V Gehrke S Adane B. Targeting glutamine metabolism and redox state for leukemia therapy. Clin Cancer Res (2019) 25(13):4079–90. doi: 10.1158/1078-0432.CCR-18-3223 PMC6642698 30940653

[55] Edwards DN Ngwa VM Raybuck AL Wang S Hwang Y Kim LC. Selective glutamine metabolism inhibition in tumor cells improves antitumor T lymphocyte activity in triple-negative breast cancer. J Clin Invest (2021) 131(4):e140100. doi: 10.1172/JCI140100 PMC7880417 33320840

[56] DeBerardinis RJ Mancuso A Daikhin E Nissim I Yudkoff M Wehrli S. Beyond aerobic glycolysis: transformed cells can engage in glutamine metabolism that exceeds the requirement for protein and nucleotide synthesis. Proc Natl Acad Sci U S A (2007) 104(49):19345–50. doi: 10.1073/pnas.0709747104 PMC2148292 18032601

[57] Huang F Ni M Chalishazar MD Huffman KE Kim J Cai L. Inosine monophosphate dehydrogenase dependence in a subset of small cell lung cancers. Cell Metab (2018) 28(3):369–82.e5. doi: 10.1016/j.cmet.2018.06.005 30043754 PMC6125205

[58] Kodama M Nakayama KI. A second warburg-like effect in cancer metabolism: The metabolic shift of glutamine-derived nitrogen: A shift in glutamine-derived nitrogen metabolism from glutaminolysis to *de novo* nucleotide biosynthesis contributes to malignant evolution of cancer. Bioessays (2020) 42(12):e2000169. doi: 10.1002/bies.202000169 33165972

[59] Kodama M Oshikawa K Shimizu H Yoshioka S Takahashi M Izumi Y. A shift in glutamine nitrogen metabolism contributes to the malignant progression of cancer. Nat Commun (2020) 11(1):1320. doi: 10.1038/s41467-020-15136-9 32184390 PMC7078194

[60] Quéméneur L Gerland LM Flacher M Ffrench M Revillard JP Genestier L. Differential control of cell cycle, proliferation, and survival of primary T lymphocytes by purine and pyrimidine nucleotides. J Immunol (2003) 170(10):4986–95. doi: 10.4049/jimmunol.170.10.4986 12734342

[61] Claiborne MD Sengupta S Zhao L Arwood ML Sun IM Wen J. Persistent CAD activity in memory CD8+ T cells supports rRNA synthesis and ribosomal biogenesis required at rechallenge. Sci Immunol (2022) 7(71):eabh4271. doi: 10.1126/sciimmunol.abh4271 35622902 PMC9307092

[62] Chang CH Qiu J O’Sullivan D Buck MD Noguchi T Curtis JD. Metabolic competition in the tumor microenvironment is a driver of cancer progression. Cell (2015) 162(6):1229–41. doi: 10.1016/j.cell.2015.08.016 PMC4864363 26321679

[63] Klein D. The tumor vascular endothelium as decision maker in cancer therapy. Front Oncol (2018) 8:367. doi: 10.3389/fonc.2018.00367 30250827 PMC6139307

[64] Amersfoort J Eelen G Carmeliet P. Immunomodulation by endothelial cells – partnering up with the immune system? Nat Rev Immunol (2022) 22(9):576–88. doi: 10.1038/s41577-022-00694-4 35288707 PMC8920067

[65] Kim B Li J Jang C Arany Z. Glutamine fuels proliferation but not migration of endothelial cells. EMBO J (2017) 36(16):2321–33. doi: 10.15252/embj.201796436 PMC5556269 28659379

[66] Mestre-Farrera A Bruch-Oms M Peña R Rodríguez-Morató J Alba-Castellón L Comerma L. Glutamine-directed migration of cancer-activated fibroblasts facilitates epithelial tumor invasion. Cancer Res (2021) 81(2):438–51. doi: 10.1158/0008-5472.CAN-20-0622 33229340

